# Supplementary material for: Filament formation by the translation factor eIF2B regulates protein synthesis in starved cells
Source: Biol Open. 2020 Jul 8;9(7):bio046391. doi: 10.1242/bio.046391 (PMC7358136; doi:10.1242/bio.046391)
Supplement: Supplementary information [file biolopen-9-046391-s1.pdf]

SUPPLEMETARY MOVIES

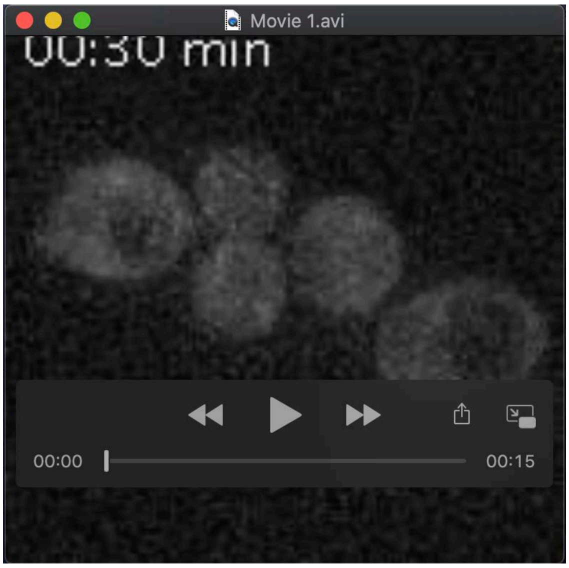

Movie 1. Filament formation by eIF2B induced by energy depletion. Live-cell time-lapse microscopy of Gcn3-sfGFP(V206R) cells grown to log phase in SC medium containing 2% glucose. The cells were then depleted of energy as described in material and methods. The movie starts at the time point of energy depletion.

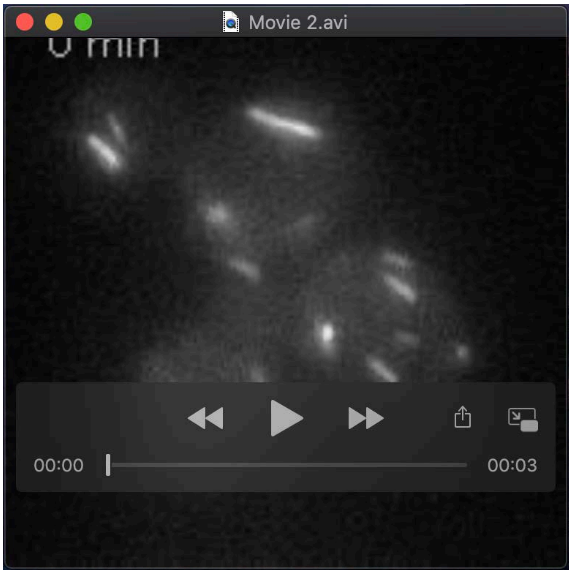

Movie 2. eIF2B filaments disassemble in less than 10 minutes. Live-cell time-lapse microscopy of Gcn3-sfGFP(V206R) cells grown to log phase in SC medium containing 2% glucose and then depleted of energy for 60 min as described in material and methods. The movie starts at the time point when cells were resupplied with SC medium containing 2% glucose.

Biology Open • Supplementary information

SUPPLEMETARY FIGURES

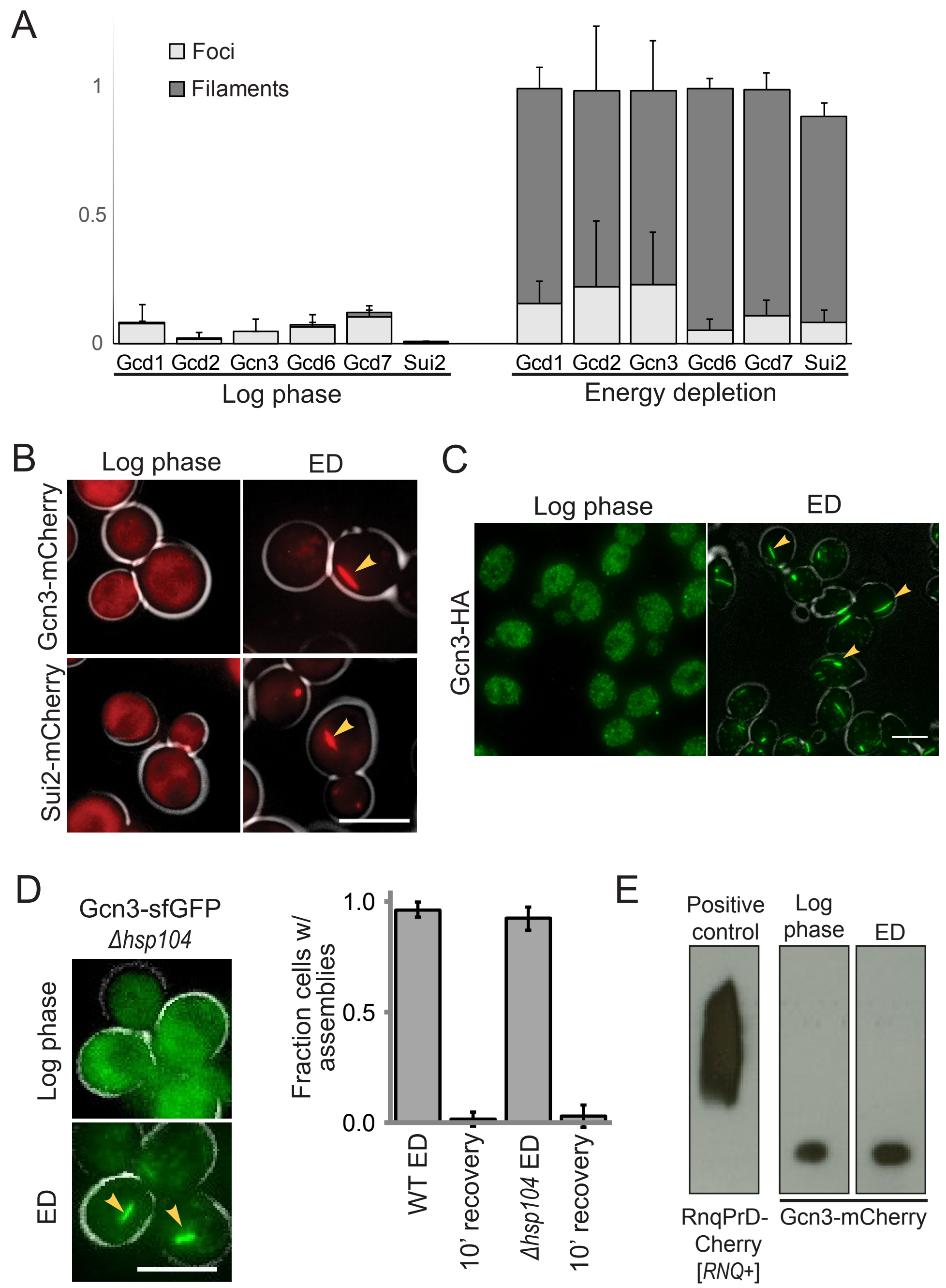

Fig. S1. Filaments are starvation-induced protein assemblies. A) Fraction of cells with eIF2B assemblies (foci – light grey; filaments – dark grey) quantified from live-cell fluorescence microscopy images. Quantification was done for cells expressing subunits of eIF2B (Gcd1p, Gcd2p, Gcn3p, Gcd6p, Gcd7p) and eIF2 (Sui2p) tagged with sfGFP(V206R) during logarithmic growth (log phase, left) and after 20 min of energy depletion (ED, right). Results were obtained from at least 200 cells per sample and 3 independent experiments. B) Sui2-mCherry and Gcn3-mCherry localization in log phase cells and after 20 min of ED. Arrows point at filaments. C) Immunofluorescence of Gcn3-HA in fixed log phase cells and after 20 min of ED. Note that the protein is diffuse in control cells and localizes in filaments in starved cells. D) Live-cell fluorescence microscopy in *HSP104* deletion background (left) and quantification of eIF2B assemblies in Δ*hsp104* and wild type cells after 1 hour of ED and 10 min after recovery in SC medium. Note that deletion of *HSP104* does not affect filament formation or dissolution. Scale bar is 5 µm. E) SDD-AGE of cell lysates from Gcn3-mCherry expressing cells from log phase (middle) and after 1 hour of ED (right). Lysates from RnqPrD-Cherry [*RNQ*+] expressing cells were used as positive control (left).

Biology Open • Supplementary information

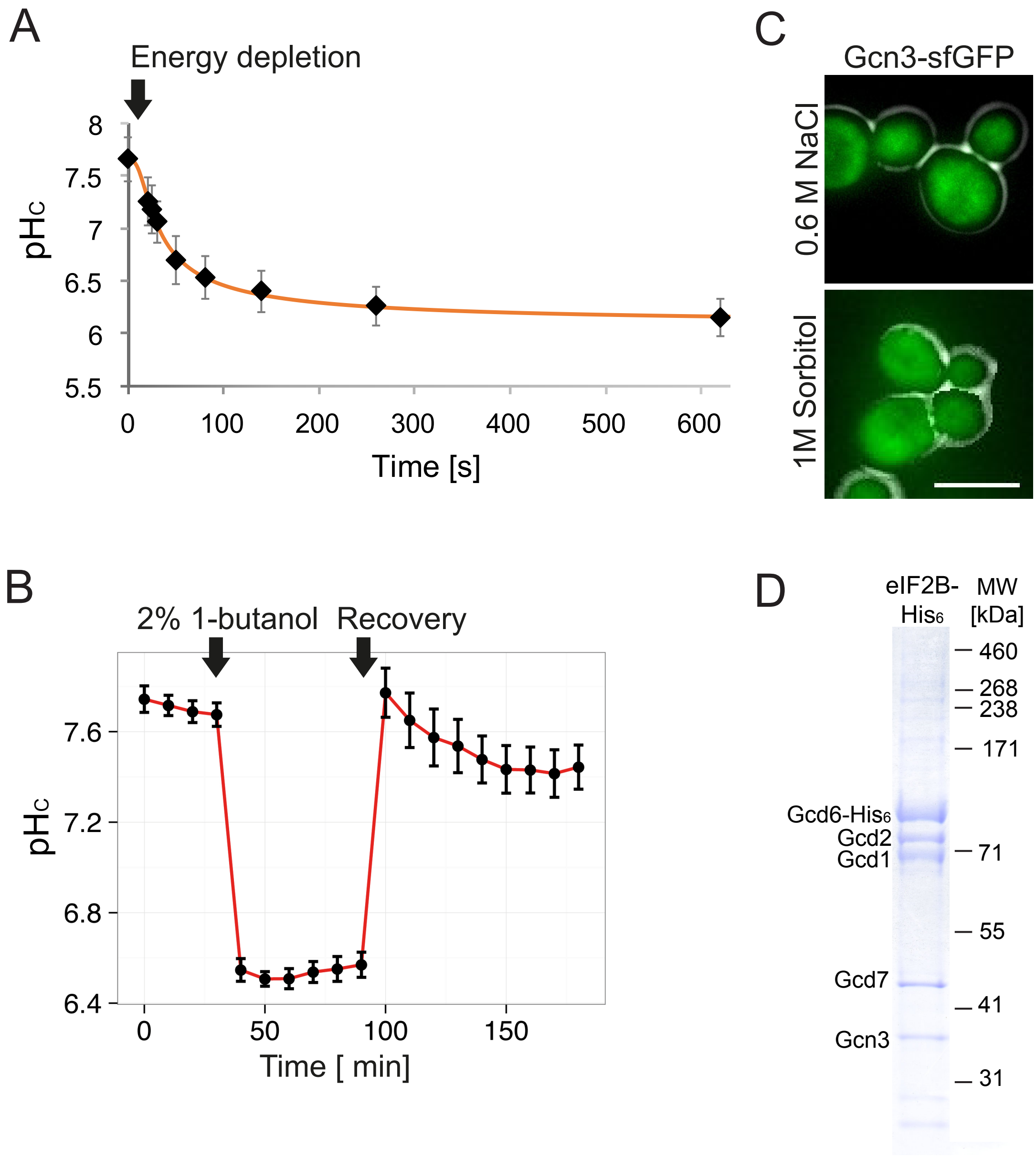

Fig. S2. Filamentation-inducing conditions cause reversible acidification of the cytosol. A) pHc changes in pHluorin2-expressing culture during energy depletion (ED) as measured by flow cytometry. Note that pHc drops by one unit within 3 min. B) pH measurement during fusel alcohol stress as determined by live-cell fluorescence microscopy of yeast expressing pHluorin2. Note that pHc drops rapidly upon addition of 2% 1-butanol and recovers to normal levels upon stress release. C) Fluorescence microscopy of Gcn3-sfGFP(V206R) expressing yeast after 20 min of treatment with 0.6 M NaCl (upper panel) and 1 M sorbitol (lower panel). Note that osmotic stress does not induce assembly formation. Scale bar is 5 µm. D) Acrylamide gel of eIF2B with His-tag after purification from insect cells. Protein bands are visualized via staining with Coomassie Brilliant Blue.

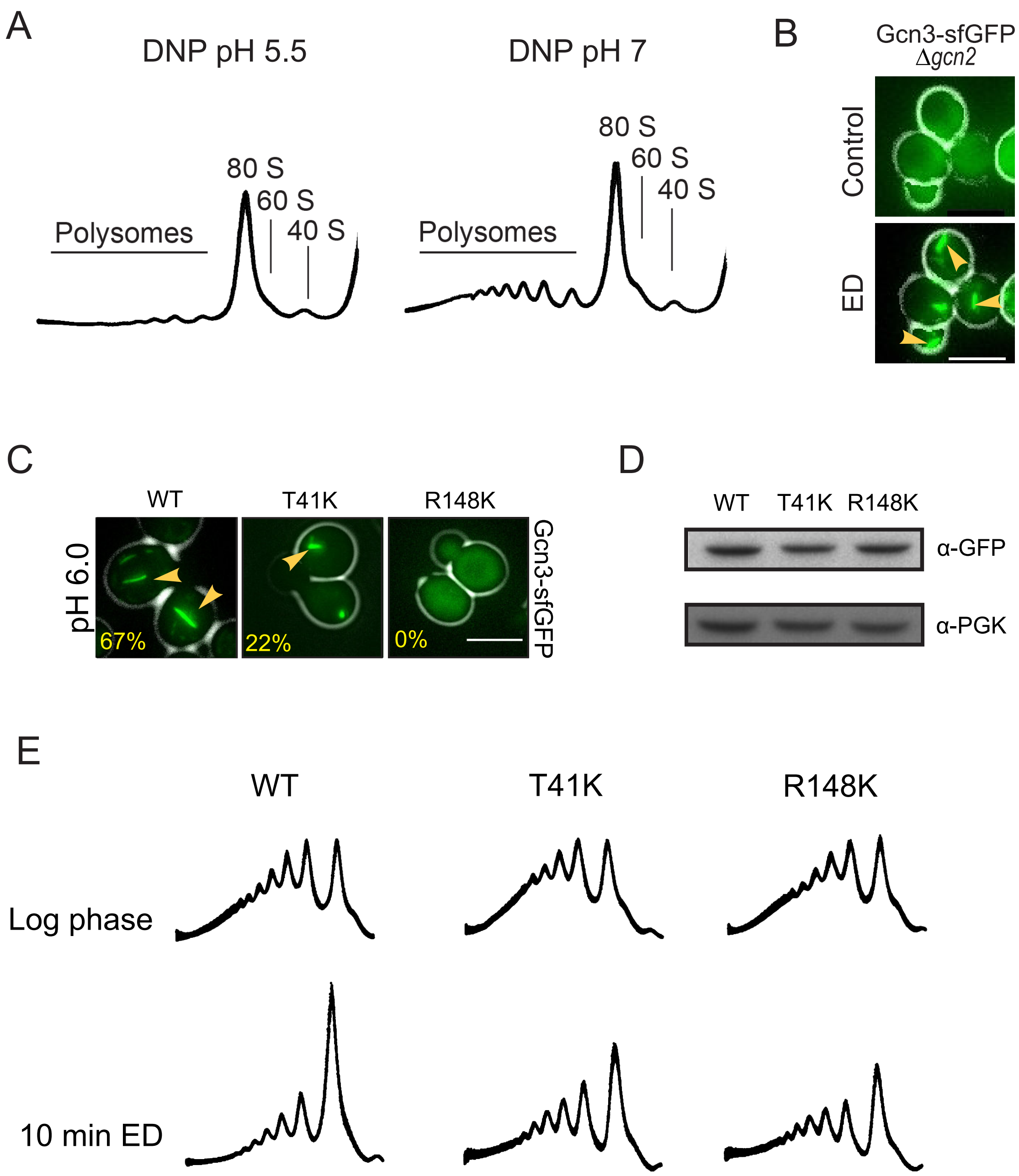

Fig. S3. Filament formation is required for efficient translation downregulation. A) Polysome profiles after 10 min of treatment with 20 mM DNP pH 5.5 (left) and pH 7.0 (right) in the presence of 2% glucose (w/v). The regions corresponding to polysomes as well as the monosome subunits are indicated. B) Live-cell fluorescence microscopy of Gcn3-sfGFP-expressing cells lacking Gcn2p. Log phase cells are shown in the upper panel and cells treated for 20 min with energy depletion (ED) are shown below. Note that Gcn2 deletion does not affect filament formation. Scale bar is 5 µm. C) Imaging of wild-type cells (WT) and mutant cells that showed filament formation deficiency during ED (T41K, R148K). Imaging was done after 20 min incubation in pH 6 buffer containing 2% glucose and 20 mM DNP. Note that T51K forms fewer filaments and no filaments form in R148K expressing cells. Scale bar is 5 µm. D) immunoblot of GFP-tagged Gcn3p from lysates of log phase cells expressing wild-type Gcn3p, T41K or R148K. Pgk1p was used as a loading control. A GFP-specific antibody and a Pgk1p-specific antibody were used for immunodetection, respectively. Note that WT, T41K and R148K protein level do not differ. E) Polysome profiles of WT, T41K and R148K lysates from cells grown to log phase (Log phase) and treated for 10 min by ED.

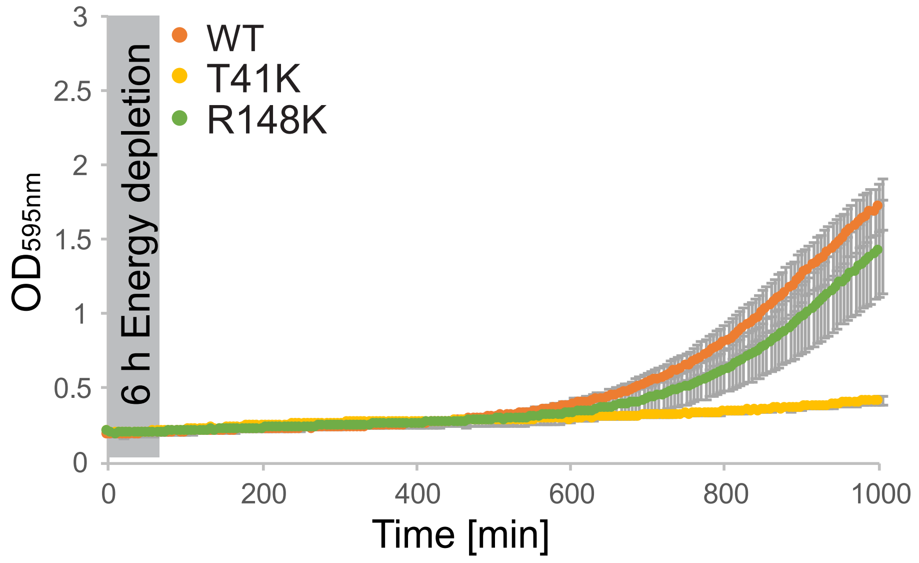

Fig. S4. **Filament formation is essential for recovery from energy depletion.** Growth curves of WT and mutant (T41K, R148K) strains diluted in SC medium to OD 0.05 after 6 hours of energy depletion. Growth was determined by measuring the optical density at 595 nm. Plots were generated from triplicates of 3 different biological replicates and the experiment was carried out at least 3 times.

Biology Open • Supplementary information

SUPPLEMENTARY TABLES

Table S1. Yeast strains used in this study.

Name/Modification	Genotype	Source/Reference
W303 *ADE+*	*MATalpha can1-100 his3-11,15 leu2-3,112 trp1-1 ura3-1*	Gift, Lindquist lab
	**Alternative name**	
W303 *ADE+ GCD1::mCherry-SpHIS5*	3663	This study
W303 *ADE+ GCD2::mCherry-SpHIS5*	3665	This study
W303 *ADE+ GCN3::mCherry-SpHIS5*	3671	Petrovska et al.
W303 *ADE+ GCD6::mCherry-SpHIS5*	3667	This study
W303 *ADE+ GCD7::mCherry-SpHIS5*	3669	This study
W303 *ADE+ SUI2::mCherry-SpHIS5*	4324	This study
W303 *ADE+ GCD1::sfGFP(V206R)- KanMX*	3661	This study
W303 *ADE+ GCD2::sfGFP(V206R)- KanMX*	3135	This study
W303 *ADE+ GCN3::sfGFP(V206R)- KanMX*	3141	Petrovska et al.
W303 *ADE+ GCD6::sfGFP(V206R)- KanMX*	3137	This study
W303 *ADE+ GCD7::sfGFP(V206R)- KanMX*	3138	This study
W303 *ADE+ SUI2::sfGFP(V206R)-KanMX*	3136	This study
W303 *ADE+ GCN3::HA-SpHIS5*	4057	This study
W303 *ADE+ gcn3-Δ*	3682	This study
W303 *ADE+ gcn3-Δ* Gcd1::sfGFP(V206R)-*SpHIS5*	3682	This study
W303 *ADE+ gcn2-Δ*	3680	This study
W303 *ADE+ gcn2-Δ* Gcn3::sfGFP(V206R)-*SpHIS5*	4127	This study
W303 *ADE+ hsp104-Δ*	3401	Gift, Lindquist lab
W303 *ADE+ hsp104-Δ* Gcn3::sfGFP(V206R)-*SpHIS5*	4325	This study
W303 *ADE+ GCN3(T41K)*	4288	This study
W303 *ADE+ GCN3(R148K)*	4291	This study
W303 *ADE+ trp::GPD-ypHluorin2 GCN3::sfGFP(V206R)-SpHIS5*	3923	Munder *et al.*
W303 *ADE+ trp::GPD-ypHluorin2 GCN3(T41K)::sfGFP(V206R)-SpHIS5*	4275	This study
W303 *ADE+ trp::GPD-ypHluorin2 GCN3(R148K)::sfGFP(V206R)-SpHIS5*	4279	This study
W303 *ADE+ GCN3(T41K)::sfGFP(V206R)-SpHIS5*	4269	This study
W303 *ADE+ GCN3(R148K)::sfGFP(V206R)-SpHIS5*	4270	This study
BY4741 [RNQ+], pAG415GPD-Rnq1LC-mCherry	9, O-1354	Kroschwald et al.

Biology Open • Supplementary information

Table S2. List of plasmids used in this study.

**Protein expression**	**Alternative name**
pBIG-GCN3-GCD7-GCD2	3031
pBIG-HIS6-GCD6- GCD1	3032
**C-terminal tagging**	
pKT128-sfGFP(V206K)	L-264
pKT127-sfGFP(V206K)	L-263
pKT128-mCherry	L-295
pYM45	L-353
**Integration of pHluorin2**	
pAG304GPD-pHluorin2	O-3192
**Gene deletion**	
pUG6	L-178
pUG27	L-179

Biology Open • Supplementary information

Table S3. List of oligos used in this study.

	Alternative name	Sequence
**C-terminal tagging**		
Gcd1	1459/1460	TATACCGACGAGTACGAGTACGAAGATGACGGATTATTTGAGCGT GGTGACGGTGCTGGTTAA/ ACTTGGTCTCTATTAAAGAGACTGAAGGAATATACATAAGTTTATA TCGATGAATTCGAGCTCG
Gcd2	1111/1112	CCTTCATCTGTCCCTGTTATTTTAAGAGAGTACAAAGGTTCCGCA GGTGACGGTGCTGGTTAA/ TGTAGTATACACACGTGGACGACCATTAACGAATGTTGTATATGC TCGATGAATTCGAGCTCG
Gcn3	1117/1118	ACTCCAAGTGCCGTTTCAGAAGAGTTAATCAAGATGTGGTATGAT GGTGACGGTGCTGGTTAA/ TCCTTTTCTATTAAGTCATTGCGTGCATATATTATGTGATTTTTT TCGATGAATTCGAGCTCG
Gcd6	1113/1114	GAGTGGTTACAGAATGCTGACGAAGAATCTTCCTCAGAAGAGGAA GGTGACGGTGCTGGTTAA/ CCTTTTTATGGTAATACATGATTATGCAGGGTACTGCGTGGACTT TCGATGAATTCGAGCTCG
Gcd7	1115/1116	GATAATTACAAGCAAATTGATGTGCATTTGGATAAAAATAAGGCG GGTGACGGTGCTGGTTAA/
Sui2	1119/1120	AATAGATCTGACTCTGAAGACGATGAGGATGAGTCAGACGACGAG GGTGACGGTGCTGGTTAA/ AGTATGACACTTGAAAACACCTAGAAAAATTAGGCGCGGCAATGA TCGATGAATTCGAGCTCG
Gcn3-HA	1117/1945	ACTCCAAGTGCCGTTTCAGAAGAGTTAATCAAGATGTGGTATGAT GGTGACGGTGCTGGTTAA/ ACTCCAAGTGCCGTTTCAGAAGAGTTAATCAAGATGTGGTATGAT CGTACGCTGCAGGTCGAC
**Gene deletion**		
Gcn3	1463/1464	TTTTCTATTAAGTCATTGCGTGCATATATTATGTGATTTTTTTTA GCATAGGCCACTAGTGGATCTG/ GAATTTTAAAAGTCCTACGTATACCAGAAATCGAGAGGAAGGATG CAGCTGAAGCTTCGTACGC
Gcn2	1461/1462	CTTTAACTGATGCGTTATAGCGCCGCACAGATCTTTAAAGGCCTA GCATAGGCCACTAGTGGATCTG/ TTTCAATAATTTTCCGTTCCCCTTAACACATACTATGTATAAATG CAGCTGAAGCTTCGTACGC
**Allele replacement**		
Gcn3	1453/1454	AATTCCAGCTGACCACCATG ATGTCGGAGTTTAATATTACA/ GATCCCCGGGAATTGCCATG TTAATCATACCACATCTTGA
Gcd1	1902/1903	AATTCCAGCTGACCACCATGTCAATTCAGGCTTTTGTC/ GATCCCCGGGAATTGCCATGTTAACGCTCAAATAATCCGT

Biology Open • Supplementary information
